# Esophageal Entrapment in a Thoracic Spine Fracture-Dislocation Injury After a Motor Vehicle Accident

**DOI:** 10.7759/cureus.63140

**Published:** 2024-06-25

**Authors:** Sarah Danehower, Emily Sieg

**Affiliations:** 1 Neurological Surgery, University of Louisville Hospital, Louisville, USA; 2 Neurosurgery, University of Louisville, Louisville, USA

**Keywords:** motor vehicle collison, vertebral fusion, thoracic spine fracture, blunt thoracic trauma, esophageal surgery

## Abstract

Thoracic spine fracture-dislocation injuries result from significant forces that cause significant morbidity and mortality. In rare instances, there have been cases of associated esophageal injury from bony laceration. Here we report a case esophageal entrapment in a high thoracic distraction injury following a motor vehicle accident.

## Introduction

Thoracic spine fracture-dislocation injuries result from significant forces that disrupt the anterior, middle, and posterior columns of the spine. They are rare and associated with significant neurologic morbidity and mortality. Additionally, these injuries are often associated with concomitant chest injuries. In rare cases, there have been reports of thoracic spine flexion-dislocation injuries with associated vertebral body fractures leading to esophageal perforation due to blunt injury [1-4]. Recovery from esophageal injuries depends on early identification and repair [3]. However, these injuries are difficult to identify due to confounding factors, thereby delaying care. Here we report a case of early identified esophageal entrapment in a high thoracic distraction injury following a motor vehicle accident.

## Case presentation

**Figure 1:**
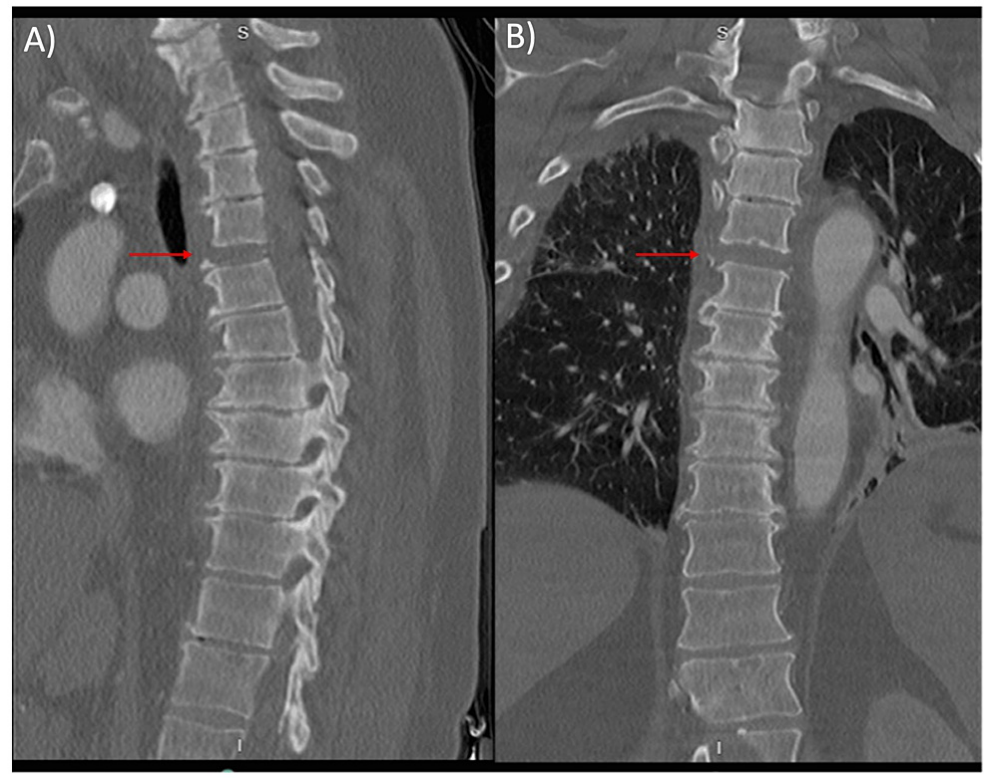
Sagittal (A) and Coronal (B) CT of Thoracic Spine T4/5 fracture-disruption with retrolisthesis of T4 on T5 as indicated with red arrows.

A 61-year-old woman presented to the emergency department (ED) as an unrestrained MVA victim. Upon presentation, she was alert and oriented, able to move all extremities but hypotensive with mean arterial pressures in the 50s. During her initial assessment in the ED, the patient was found to have active extravasation from a significant liver laceration on ultrasonography. The patient was taken emergently to the operating room by general surgery for definitive repair and remained intubated and sedated afterward. Following stabilization, the patient underwent further imaging studies. On computed tomography (CT) scan she was noted to have a thoracic 4/5 distraction injury (Figure 1).

**Figure 2:**
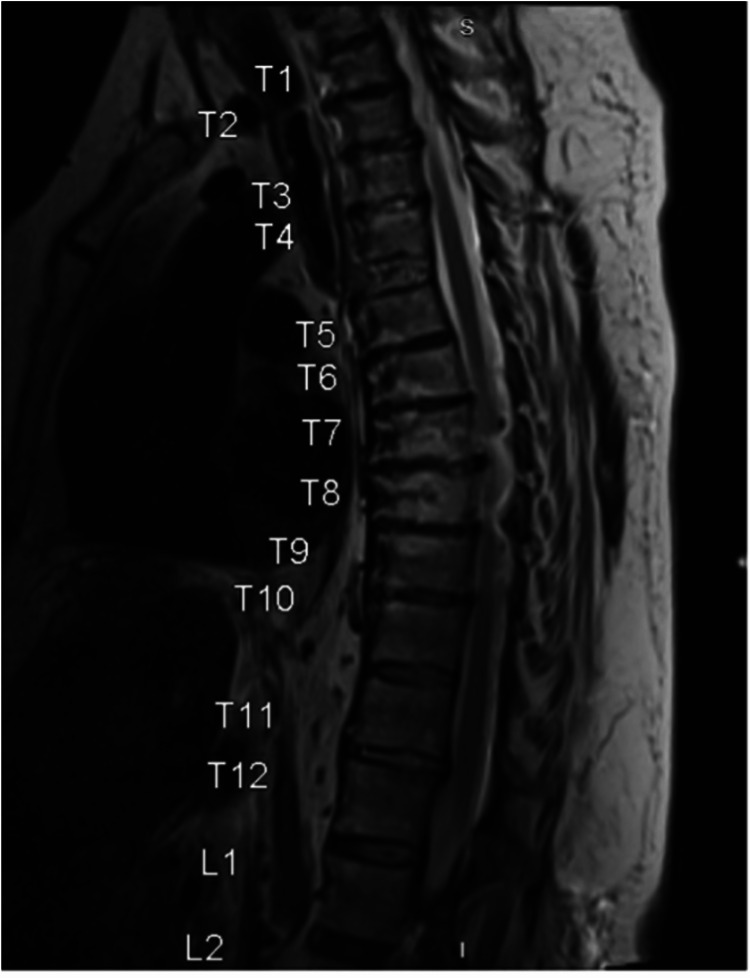
T2-weighted sagittal MRI T4/5 disc disruption as well as anterior longitudinal ligamentous and ligamentum flavum disruption without spinal cord injury

The patient was intubated and sedated on the initial neurological exam but able to move both lower extremities antigravity to noxious stimuli. Magnetic resonance imaging (MRI) of the thoracic spine was obtained, which confirmed a three-column ligamentous injury and disc disruption at T4/5 without spinal cord injury (Figure 2).

**Figure 3:**
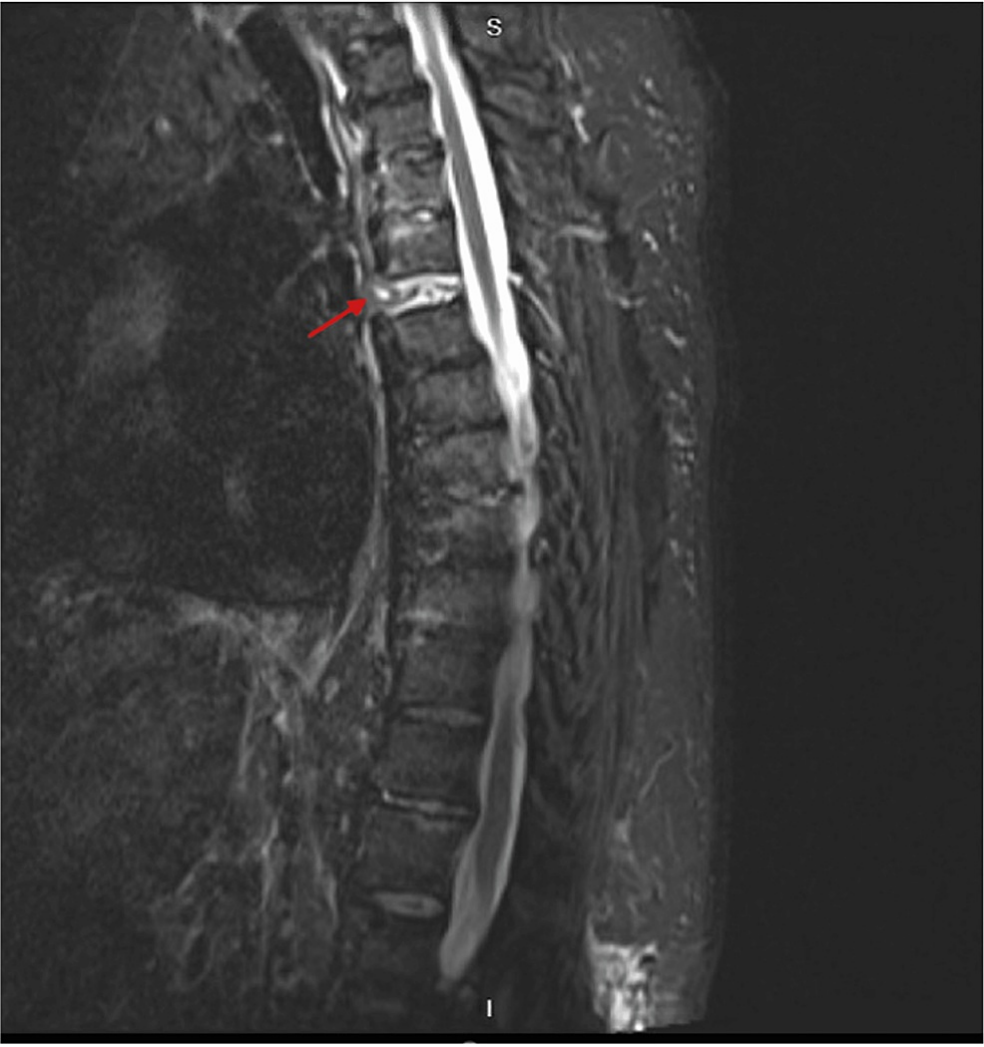
T2-STIR sagittal MRI Esophageal herniation into T4/5 disrupted disc space as indicated by arrow. STIR: short T1 inversion recovery

MRI T2 short T1 inversion recovery (STIR) sequence further revealed esophageal herniation into disrupted disc space at the level of the injury (Figure 3).

General surgery performed an esophagogastroduodenoscopy (EGD) to rule out esophageal injury. EGD confirmed retropulsion of the esophagus into the spine at 23cm without perforation or injury. To reduce the esophageal herniation, the general surgery team elected to place an esophageal stent. Following stent placement, posterior thoracic instrumentation of T2 to T7 with pedicle screw fixation and fracture reduction was performed. Pedicle screws for T4 and T5 on the left side were aborted given the patient's small pedicles and inability to safely place screws. Following this procedure, an EGD was again performed which confirmed no esophageal injury after thoracic spine fixation. Postoperatively, the patient recovered from surgery. Given her extensive traumatic injuries and airway edema, the patient was unable to wean from a ventilator. She required a tracheostomy and percutaneous gastrostomy tube, and clinical assessment for esophageal injury was unable to be obtained during hospital admission.

## Discussion

Esophageal injury is a rare phenomenon in the trauma setting. Roughly 10% of esophageal injuries are due to penetrating or blunt injury while the remaining 90% are due to iatrogenic causes or perforation by foreign bodies [5-8]. Esophageal injury in the setting of thoracic spine trauma is exceedingly rare but has been reported multiple times in the literature. However, the diagnosis of esophageal injury secondary to blunt injury from thoracic spine trauma is often missed or delayed, resulting in significant patient morbidity. Maroney et al. were the first to report esophageal entrapment after thoracic spine fracture-dislocation [9]. In this case report, the entrapment was only revealed after CT with oral contrast was conducted for persistent dysphagia. Imaging revealed contrast extravasation into the mediastinum. Given the delay in esophageal injury, the patient suffered an infection and ultimately required thoracotomy with diverting esophagostomy, gastrostomy tube placement, and mediastinal drain for contamination.

DeLappe et al. report a case of esophageal entrapment that was discovered after a patient re-presented to the hospital with complaints of dysphagia and nausea after being discharged four days prior [3]. Given the delay in the identification of esophageal injury, the patient suffered an esophageal hematoma and edema necessitating gastrostomy tube placement.

## Conclusions

Identification of esophageal injury after thoracic spine trauma remains a difficult and costly problem. Key signs of esophageal injury include pneumomediastinum, subcutaneous emphysema, pneumothorax, or pleural effusion. However as seen in the literature, injuries may be occult on initial CT imaging. Furthermore, given the neurologic morbidity of thoracic fracture dislocations, patients may lack the ability to properly display or communicate signs and symptoms of esophageal injury. Despite the rarity of blunt esophageal injuries from thoracic fracture-dislocations, careful attention should be paid to ruling out esophageal injury prior to any neurosurgical instrumentation given the close proximity of the esophagus to the anterior spinal column. The increasing use of MRI in the setting for fracture-dislocation injuries may allow for earlier identification of these injuries, allowing for earlier EGD with stent placement and prevention of iatrogenic injury.
